# The Prebiotic Diet: Other Dietary Molecules Implicated in Gut Microbiota Health

**DOI:** 10.3390/foods13030490

**Published:** 2024-02-03

**Authors:** Emanuele Rinninella, Lara Costantini

**Affiliations:** 1 UOC di Nutrizione Clinica, Dipartimento di Scienze Mediche e Chirurgiche Endocrino-Metaboliche, Fondazione Policlinico Universitario A. Gemelli IRCCS, 00168 Rome, Italy; emanuele.rinninella@unicatt.it; 2 Dipartimento di Medicina e Chirurgia Traslazionale, Università Cattolica del Sacro Cuore, 00168 Rome, Italy; 3 Department of Ecological and Biological Sciences (DEB), Tuscia University, 01100 Viterbo, Italy

In 2016, the International Scientific Association for Probiotics and Prebiotics (ISAPP) provided a new definition of a prebiotic as “a substrate that is selectively utilized by host microorganisms conferring a health benefit”. Moreover, they added polyunsaturated fatty acids and phenolics/phytochemicals among the prebiotics, even though they are considered ‘candidates’ due to the different levels of scientific evidence in comparison to accepted prebiotics such as fructans and galactans [1]. However, it is not to be excluded that there may be dietary molecules that could play a fundamental role in promoting commensal bacteria viability and metabolic activity, and conferring health benefits to the host, even though they are not substrates for them, and instead, other dietary molecules that interrupt or inhibit these metabolic activities. For this reason, we had previously introduced the concept of the “correct prebiotic diet” [2], indicating that bacteria, like all living organisms, cannot benefit from a single carbon source but rather require a series of molecules and metabolites which can also be produced by other bacterial strains, resulting in an interdependence between them as well as with the host. We had previously given an example of this interdependence, pointing to the ability of some Lactobacillaceae to metabolize the omega-6 fatty acid linoleic acid (LA) into 10-hydroxy-*cis*-12-octadecenoic acid (HYA), a much healthier compound in comparison to its precursor. Indeed, HYA shows anti-inflammatory activity against systemic inflammation, and this is in opposition to the inflammatory activity promoted by LA [2]. Western diets are known to be high in omega-6 fatty acids and low in healthier omega-3 fatty acids, so it could be speculated that gut bacteria are attempting to restore this imbalance by turning LA into HYA.

Meanwhile, in the literature, there is increasing evidence of correlations between unhealthy eating habits, pathologies, and intestinal dysbiosis. In this collection, in the paper by Al-Musharaf et al. [3], an inverse correlation between the richness and diversity of the gut microbiota and low serum vitamin B_12_ levels was found in obese Saudi females. In this study, the authors highlighted that obese volunteers with low serum B_12_ also had a specific gut microbiota, and this gut microbiota is different from that of lean volunteers with normal serum vitamin B_12_ levels [3]. Although a causal relationship was not determined between B_12_ deficiency, dysbiosis, and obesity, it is believed that there is a bidirectional correlation, particularly considering that vitamin B_12_ is both a metabolic cofactor of the microbiota when the vitamin comes from the diet and is also a product of bacterial synthesis. Therefore, B_12_ deficiency could trigger dysbiosis, which could lead to obesity [3].

What is certain is that the correct diet is closely related to the maintenance of a healthy gut microbiota, and both the diet and microbiota contribute to protection against the onset of other pathologies. This is also evident during pregnancy, where the combination of bioactive compounds and probiotics allows for, in addition to the maintenance of the maternal commensal microbiota, proper infant gut microbiota colonization. This condition, in addition to preventing the onset of obesity, gestational diabetes, gestational hypertension, and preeclampsia in the pregnant woman, may prevent allergies, colic, and other maladies in newborns [4].

However, as is well known, the concept of an incorrect diet is not only confined to the phenomenon of nutritional deficiencies; on the contrary, it is increasingly linked to energy surplus. Therefore, similarly, dysbiosis due to incorrect diet could occur not only in conditions of deficiencies of useful metabolites but also in the presence of an excess of toxic metabolites. In this context, the review of Mutalub et al. [5] focused attention on the gut microbiota metabolites related to the onset of cardiometabolic diseases. Among these, trimethyl amine-N-oxide (TMAO) is produced by the gut microbiota as a result of choline and carnitine metabolism from a red meat-rich diet. High TMAO levels are a risk factor for peripheral artery disease, myocardial infarction, stroke, and heart failure, and a specific TMAO-generating dysbiotic gut microbiota composition is present in the gut of subjects with high plasmatic TMAO levels. Among these, there are *Prevotella*, *Akkermansia*, *Ruminococcus gnavus*, *Sporobacter*, Pseudomonadota, Bacillota, and Actinomycetota. Moreover, the same Western dietary pattern can provide a further substrate to the gut microbiota, which results in the production of an additional toxic metabolite. This is the case for urea, which can be transformed into uremic toxins by the ureases of the gut bacteria. Uremic toxins directly lead to adverse cardiac remodeling by stimulating the cardiac fibroblasts and collagen synthesis [4]. The authors pointed out that multiple approaches including the modulation of the diet, for example, diets low in red meat (i.e., those low in carnitine/choline and urea, such as the Mediterranean diet) combined with a precise modulation of the microbiota through probiotics, could, in this case, be part of personalized medicine interventions aimed at the prevention of cardiometabolic diseases [5]. 

Growing evidence demonstrates that dietary polysaccharides, coming from a high-fiber diet (HFD) such as the Mediterranean diet, allow for the maintenance of the eubiosis of the gut microbiota, preventing several pathologies [6]. Considering that epidemiological evidence highlights that adherence to HFD in people is increasingly lower, especially in Western countries, dietary strategies such as the use of dietary supplements based on soluble and insoluble fibers are constantly evaluated in the literature as a real approach for the prevention and treatment of the metabolic disorders [6]. With this purpose, a new trend of obtaining prebiotic fibers from agri-food by-products is emerging from the literature, to reduce environmental impact from the perspective of the circular economy. In this collection, the paper of Valladeres-Diestra et al. [7] reviewed the possibility of obtaining xylooligosaccharides (XOs; i.e., non-digestible oligosaccharides with high prebiotic potential) from lignocellulosic biomasses. This strategy could encourage the production of XOs, making it more economically advantageous. Anyway, some challenges remain to be resolved in producing XOs functional prebiotics; we still need to consolidate knowledge on their mechanism of action, their useful and non-toxic doses, and their stability over time [7]. With a similar scope, the paper of Bonifacio-Lopes et al. [8] investigated the prebiotic activity of brewer’s spent grain, a food by-product from the brewing industry. The authors investigated the probiotic activity of this matrix on human fecal microbiota; growth of *Lactobacillus* spp. and *Bifidobacterium* spp. was found, along with significant production of short-chain fatty acids, suggesting a positive prebiotic action of this by-product [8].

Finally, some molecules have more recently become part of our diets, which could perturb the activity and composition of the gut microbiota. For example, in Western diets, many novel agents are used in processed foods, whose interaction with the microbiota is almost unknown. This is the case for food emulsifiers addressed in this collection [9], which by their chemical nature (i.e., consisting of both hydrophilic and hydrophobic parts) can increase the permeability of the membranes determining the translocation of the lipopolysaccharide and the onset of low-grade inflammation. Moreover, emulsifiers are non-adsorbed compounds that can directly interact in the lumen with the gut microbiota, changing its composition or activity. Initial evidence has shown that food emulsifiers can induce microbiota dysbiosis through a more inflammatory microbial composition. However, it is necessary that we understand the relationships that these molecules could have with the metabolism of other molecules in our gut lumen; in this area, studies are still lacking. For example, early studies have highlighted that an emulsifier known by the name of Polysorbate 80 can cause the absorption of phthalate, a plastic contaminant used in food packaging, thereby inducing the loss of gut membrane continuity. Considering the high quantity of products packaged in plastic agents and the high quantity of emulsifiers present in industrial foods, the impact of these mechanisms on our health could be very significant [9].

In conclusion, the following collection further highlighted the intricate interactions that occur between diet, microbiota, and pathologies, especially metabolic ones. However, the relationships between the different bacterial populations, the microbiota’s metabolic action on different dietary compounds, and the actions of the microbiota’s secondary metabolites in humans still must be fully understood, and these questions should be the new frontiers in the field of gut microbiota research. 

## Data Availability

Data sharing is not applicable.
